# Algorithm-based analysis of lymph node dissection strategies and survival outcomes in primary oral squamous cell carcinoma

**DOI:** 10.3389/fonc.2025.1483921

**Published:** 2025-04-16

**Authors:** Andreas Vollmer, Babak Saravi, Alexander Kübler, Urs Müller-Richter, Anna Winter, Simon Nagler, Marius Hörner, Sebastian Gubik, Stefan Hartmann

**Affiliations:** ^1^ Department of Oral and Maxillofacial Plastic Surgery, University Hospital of Würzburg, Würzburg, Germany; ^2^ Department of Orthopedics and Trauma Surgery, Medical Center - University of Freiburg, Faculty of Medicine, University of Freiburg, Freiburg, Germany; ^3^ Department of Prosthodontics, Julius Maximilian University Würzburg, Würzburg, Germany

**Keywords:** head and neck neoplasms, neck dissection, lymph node yield, survival rate, Kaplan-Meier estimate, proportional hazards model

## Abstract

**Introduction:**

Recent advancements in treatment approaches for oral squamous cell carcinomas (OSCCs) necessitate a reevaluation of neck dissection techniques and their impact on patient outcomes and morbidity.

**Methods:**

This retrospective study of 250 OSCC patients recruited between 2017–2022 examined the association between neck dissection techniques and survival metrics. Our cohort, drawn from a primary OSCC surgery population at our clinic, provided a rich dataset encompassing demographics, clinical parameters, and detailed surgical records. Two neck dissection techniques were analyzed: the Supraomohyoid Selective Neck Dissection (SND), which targets lymph nodes at Levels I–III, and Other Dissections (OD), which involve a more extensive extraction including Levels IV and V. Kaplan-Meier survival curves and Cox proportional hazards models assessed the influence of lymph node dissection on postoperative outcomes.

**Results:**

Findings indicated that each additional lymph node removed was associated with a 0.289-day increase in hospitalization (p = 0.002), yet no significant link was found between dissection techniques or total lymph node extraction count and survival metrics. Levels I to III emerged as critical areas with the highest likelihood of yielding tumor-positive lymph nodes, emphasizing the significance of these levels.

**Discussion:**

The study suggests that more extensive dissection does not necessarily confer survival benefits, highlighting the importance of strategic surgical focus and the potential for tailored interventions that prioritize disease-specific lymph node levels to optimize patient recovery and prognosis.

## 1 Introduction

Oral squamous cell carcinomas (OSCCs) represent a prevalent form of carcinomas affecting the head and neck region, originating from the mucosal epithelium. Notably, the oral cavity, pharynx, and larynx constitute the primary sites for head and neck squamous cell carcinomas (HNSCC) development (1). The management of OSCCs entails a spectrum of multimodal treatment approaches upon tumor location and stage, encompassing definitive surgery, surgery with adjuvant therapy, or adjuvant therapy as a standalone option (1, 2). Given the potential for significant impacts on patient quality of life, mortality, and healthcare costs, OSCC treatment warrants careful consideration (1).

Surgical resection coupled with reconstruction through microvascular grafting or local plastic procedures constitutes a primary intervention, further tailored based on factors such as tumor stage, resection extent, and lymph node involvement (1). Depending on the individual case characteristics, adjuvant radiation therapy or concurrent chemoradiation (CRT) may be employed to enhance treatment efficacy and improve overall survival outcomes (3). Chemotherapeutic interventions, including cisplatin, targeted therapies like cetuximab, or checkpoint inhibitors like pembrolizumab already play pivotal roles in the treatment of OSCC. Especially Checkpoint-inhibitors are gaining prominence nowadays mostly in the recurrent and metastatic setting on the one handy and are already in discussion about neoadjuvant therapy regimes (4). Despite the surgical and non-surgical therapy options, long-term outcomes remain suboptimal and require further improvement. The yield of lymph nodes during lymph node dissection can serve as a quality marker in relation to the survival rate (5–7). Foo et al. demonstrated that for colon carcinomas, a yield of 20 or more lymph nodes is associated with improved survival (8). Similarly, Rosenberg et al. reported that breast cancer patients with a yield of 20 lymph nodes also exhibited better survival outcomes (9).

There has been a shift in the field of neck dissection techniques from comprehensive to selective removal of lymph nodes (10–12). The determination of neck dissection levels is influenced by tumor specifics, location and extent, yet a standardized guideline for the optimal lymph node count remains elusive (13). Consequently, variability persists depending on in-house protocols, surgeon experience, and technique (13–15). An emphasis on positive lymph node yield, which represents the percentage of positive lymph nodes on all extracted lymph nodes, has emerged as a potential quality marker for neck dissection (13, 16–18).

However, a nuanced evaluation is necessary, as relying solely on positive lymph node yield, could disproportionately reflect surgical technique (19). The interplay between patient-specific parameters in combination with positive lymph node yield, lymph node levels, and neck dissection techniques, particularly their correlation with overall survival and recurrence, presents an unexplored avenue in OSCC research. While previous research has examined various parameters impacting patient outcomes post-resection in the head and neck region (20–24), there exists a gap in understanding the context of positive lymph node yield, involved neck dissection levels and specific perioperative morbidity outcomes, such as duration of hospital stay, as well as overall survival and recurrence-free survival.

The present study seeks to bridge this gap by evaluating how the involved neck dissection levels and associated lymph node yield parameters influence overall survival and the likelihood of recurrence in OSCC patients. Additionally, this investigation employs a machine learning algorithm based on the Cox Proportional-Hazards model to analyze survival data and identify key prognostic factors. By integrating this algorithmic approach, the study provides valuable insights into refining neck dissection strategies and enhancing patient prognosis. As the landscape of OSCC management continues to evolve, understanding the intricate interplay between surgical techniques in combination with adjuvant therapies as a multivariant therapy concept and surgical outcomes assumes paramount importance.

## 2 Materials and methods

### 2.1 Study design

In this investigation, we employed a retrospective methodology, adhering to the STROBE guidelines relevant for observational studies (25). Ethical approval was granted by the Institutional Review Board at the University of Würzburg Medical Center in Germany (approval number 2022063001). Data from 250 consecutive patients, accrued between 2017 and 2022, were systematically reviewed.

The study included adult patients diagnosed with primary OSCC and treated within the specified time period frame at our clinic. To qualify for inclusion, participants were required to have a primary tumor and to have undergone initial surgery in our facility. We excluded cases presenting with T0 or Tis disease with prior biopsy, those with distant metastases (M+), and cases with incomplete (R1) resection from our analysis to ensure a more uniform cohort and avoid confounding factors related to negligible tumor burden, advanced metastatic status, and inadequate primary tumor management, respectively. To ensure a homogenous patient cohort, we focused on including patients with primary diagnoses who had not received prior treatment for this condition. Patients who had previously undergone surgery were excluded as they may present altered anatomical conditions, potentially affecting lymph node yield in the neck regions. Furthermore, we currently lack conclusive data on whether any form of prior medication or radiation therapy impacts lymph nodes at the cervical levels. While this is an intriguing question, it falls outside the scope of the present study and would require a larger patient cohort for future investigation. It is worth noting that the majority of patients treated at our clinic presented with a primary diagnosis, which aligns with the focus of our study.

While these exclusions may limit the generalizability of our results to all OSCC patients, particularly those with recurrent disease or prior treatments, they allowed us to generate a more homogenous dataset. This ensures more robust and precise analyses of the surgical and pathological variables under investigation.

### 2.2 Data handling

Patients’ medical histories were sourced from electronic medical records (EMRs). To collate the cohort, we identified individuals treated in our facility using our internal medical control system, indexed by the clinic’s organizational unit. Additionally, specific ICD (International Classification of Diseases) codes delineating malignant neoplasms of the head and neck, spanning C01 to C14.8, were referenced. Data were aggregated in a pseudonymized table format.

Parameters extracted included age, gender (male or female), body mass index (BMI), insurance classification (private or public), duration of hospital stay (quantified in days), surgery date, most recent status check date, and last-known patient status (disease-associated death, recurrence, or right-censored). Other parameters encompassed operational duration, the American Society of Anesthesiologists risk score (ASA), transplant or reconstructive procedures employed (either microvascular or non-microvascular/local flap), adjuvant therapy, TNM classification, total lymph nodes extracted, count of positive lymph nodes identified, the percentage of positive lymph nodes (positive lymph node yield), and the ischemia time of the microvascular flap. Adjuvant therapy in our cohort was exclusively limited to radiochemotherapy, administered based on the tumor stage and nodal involvement. Two neck dissection types were evaluated: The “Supraomohyoidal Selective Neck Dissection” (SND) group included cases where lymph nodes at Levels I to III were resected, without any extractions at Levels IV and V. This classification aligns with the typical definition of a Supraomohyoidal neck dissection, which targets lymph nodes located above the omohyoid muscle, primarily affected in certain types of head and neck cancers. The “Other Dissections” (OD) group included all other dissection patterns, which involve extractions at Levels I-V. This group represents more extensive dissection practices, including Levels IV and V.

### 2.3 Statistical analysis

#### 2.3.1 Explorative analyses, pairwise comparisons and survival analysis

All statistical analyses were conducted using Python and SPSS software (Version 27, IBM Corp., Armonk, NY, USA). Initially, both descriptive and exploratory statistics were generated. Continuous variables are presented as mean ± standard deviation, or as median with range, unless stated otherwise. Categorical variables are reported as counts and percentages. The Shapiro-Wilk test was employed to evaluate the normality of the distribution of continuous variables. Pairwise comparisons were conducted using either parametric or non-parametric tests, as determined by preliminary exploratory analysis. Correlation analyses were performed using Spearman’s rank correlation coefficient. Kaplan-Meier survival curves and Cox proportional hazards models were used to assess the impact of study variables on overall survival and recurrence-free survival. The log-rank test was applied for survival curve comparisons. A p-value of ≤ 0.05 was considered indicative of statistical significance.

#### 2.3.2 Cox proportional-hazards machine learning model

The machine learning analysis was done using the CoxPHSurvivalAnalysis from sksurv.linear_model in Python. K-fold cross-validation (k=5) was used to evaluate the performance of the model. The evaluation metric used was the concordance index (c-index), which measures the ability of a model to correctly rank the survival times of patients. The c-index was calculated using the “concordance_index_censored” function from the sksurv.metrics library. The event of interest, defined as either ‘Death’ or ‘Recurrence’, was encoded into a Boolean representation, mapping onto a new column labeled Event. The time to event or censoring was calculated as the number of days between Day of surgery and Day of last Report and stored in a new column named Time.

We prepared two separate target variables to facilitate separate survival analyses for ‘Death’ and ‘Recurrence’. Categorical variables T and N were encoded into ordered numerical labels. This encoding was manually specified, taking into account the clinical staging nomenclature. For instance, ‘T’ was mapped to an ordered sequence starting from ‘0’ to ‘4b’. Missing data in the column Ischemia-Time were addressed using Multiple Imputation, leveraging the Iterative imputer from scikit-learn. This model-based imputation approach estimates the missing value based on the observed values in an iterative manner until the algorithm converges.

For each fold, the model was trained on a training set and evaluated on a test set, reporting the c-index as the measure of model performance. Upon completing the 5-fold cross-validation, the coefficients of the Cox Proportional-Hazards model were extracted to serve as a measure of feature importance. These coefficients, which are log hazard ratios, indicate the risk associated with each feature. For visualization purposes, only coefficients with positive values were considered. A bar plot was generated, using the coefficients as the y-axis values, labeled as ‘Log Hazard Ratio or CoxPH Coefficients’.

## 3 Results

### 3.1 Descriptive statistics

The study cohort comprised a total of 250 patients with distinct demographic, clinical, and treatment-related characteristics (**Table 1**). To ensure comparability within the cohort, the inclusion period was restricted to 2017–2022, coinciding with the implementation of the 8th edition of the American Joint Committee on Cancer (AJCC) TNM classification for oral cavity cancer. This updated classification introduced significant modifications aimed at improving prognostic accuracy and clinical utility. Among the most notable changes was the incorporation of depth of invasion (DOI) into the T category, reflecting its critical role in assessing the risk of nodal metastasis and poor outcomes. The T classification now accounts for both tumor size and DOI, addressing limitations of earlier editions that relied solely on size. Additionally, the 8th edition introduced extranodal extension (ENE) into the N staging, a factor associated with worse prognosis due to the spread of cancer beyond the lymph node capsule. This revision allows for more precise differentiation between nodal stages and better reflects disease severity. These updates do not apply to p16-positive oropharyngeal cancer and nasopharyngeal cancer, which follow distinct staging criteria due to their unique biological and clinical behavior. Restricting the study period to align with these changes ensures consistency and enhances the reliability of cohort-based analyses (26, 27).

**Table 1:** Characteristics of the cohort.

Variable	Mean ± Std
BMI	26.03 ± 4.83
LOS	19.31 ± 11.56
Duration of operation	387.9 ± 161.58
Ischemia time	51.36 ± 27.61
Age	64.54 ± 12.26
Sex	
Male	144 (57.6%)
Female	106 (42.4%)
Insurance	
Public	208 (83.2%)
Private	42 (16.8%)
Status	
Alive	190 (76.0%)
Alive with Recurrence	19 (7.6%)
Dead	41 (16.4%)
ASA	
1	11 (4.4%)
2	165 (66.0%)
3	72 (28.8%)
4	2 (0.8%)
Transplant	
Microvascular	189 (75.6%)
Local flap	61 (24.4%)
Adjuvant therapy	
no	101 (40.40%)
yes	149 (59.60%)
T	
1	65 (26.0%)
2	107 (42.8%)
3	40 (16.0%)
4	38 (15.2%)
N	
0	176 (70.4%)
1	30 (12.0%)
2a	8 (3.2%)
2b	19 (7.6%)
2c	4 (1.6%)
3a	40 (16.0%)
3b	12 (4.8%)
L	
0	222 (88.8%)
1	28 (11.2%)
V	
0	241 (96.4%)
1	9 (3.6%)
Pn	
0	206 (82.4%)
1	44 (17.6%)
G	
0	1 (0.4%)
1	31 (12.4%)
2	156 (62.4%)
3	61 (24.4%)
4	1 (0.4%)

BMI, Body-Mass-Index; ASA, American Society of Anesthesiologists risk classification score; LOS, length of hospital stay; T, refers to the size or extent of the primary tumor; N, refers to the involvement of nearby lymph nodes; L, refers to the level of lymphatic invasion; V, refers to the level of venous invasion; Pn, refers to the presence or absence of perineural invasion; G, refers to the tumor grade; Std, standard deviation.

The average age of the patients was 64.54 ± 12.26 years. Regarding gender distribution, 57.6% of the patients were male, and 42.4% were female. The average Body Mass Index (BMI) for the sample was 26.03 ± 4.83 kg/m². The mean length of stay (LOS) was 19.31 ± 11.56 days. The average operation time (Op-Time) was 387.9 ± 161.58 minutes, while the mean ischemia time was 51.36 ± 27.61 minutes.

A vast majority (83.2%) had public insurance, while 16.8% had private insurance. In terms of survival status at last follow-up, 76.0% were alive, 7.6% were alive with recurrence, and 16.4% had died. For the ASA score, most patients were classified as ASA 2 (66.0%), followed by ASA 3 (28.8%). Regarding the type of transplant, 75.6% underwent microvascular reconstruction, and 24.4% received a local flap. Adjuvant therapy was administered to 59.6% of the cohort.

In this cohort, 42.8% of patients presented with a T2 tumor stage, and 70.4% showed no lymph node involvement (N0). Because patients with distant metastases were excluded, all cases were M0. With respect to other pathological variables, 11.2% had lymphatic invasion (L1), 3.6% had venous invasion (V1), and 17.6% had perineural invasion (Pn1). The most common tumor grade was G2 (62.4%), indicating a moderately differentiated carcinoma. Overall, these data reflect a cohort with predominantly mid-range disease severity, in which the majority presented with early T and N0 stages and underwent curative (R0) resection.

### 3.2 Trends in lymph node extraction and neck dissection types

**Figure 1:**
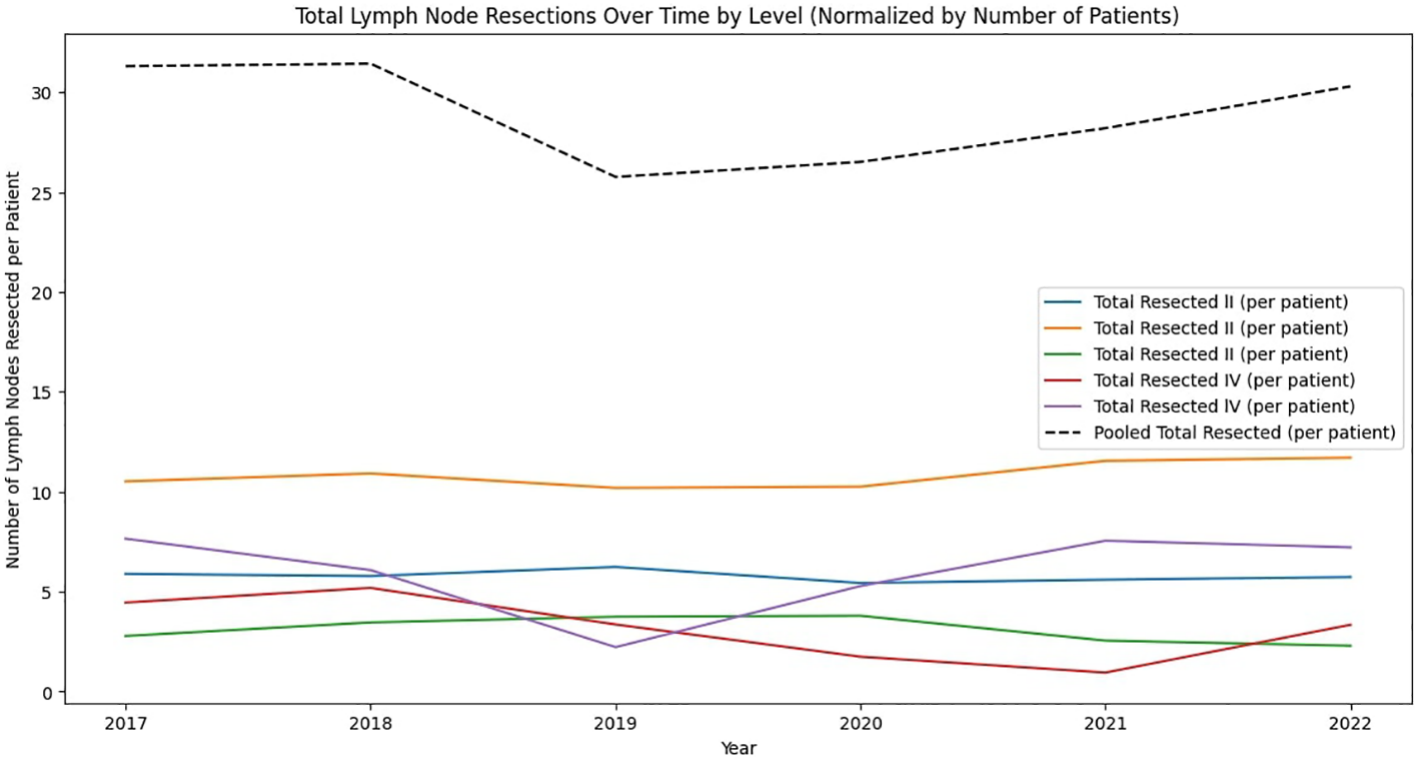
Analysis of Lymph Node Extractions by Level. This figure displays the average numbers extracted and found positive, and the positivity ratios for each anatomical level (I to V). Graphs illustrate the extent of node extraction and cancer involvement across levels, with Level I showing the highest positivity ratio, indicating a greater incidence of cancer positivity relative to nodes examined.

In our lymph node analysis across Levels I to V, Level II yielded the highest number of extracted lymph nodes (2,682), of which 47 (1.75%) tested positive for malignancy. Level I, despite having fewer total extracted lymph nodes (1,462), exhibited the highest positivity rate (2.94%, 43 positive nodes), followed closely by Level III at 2.75% (22 out of 800). Positivity rates continued to decline at higher levels, with Level IV showing 1.65% (14 out of 846) and Level V the lowest at 0.73% (10 out of 1,379). Examining per-patient averages, Level V had the highest mean number of lymph nodes extracted (12.54), whereas Level III had the lowest (4.04). By contrast, the average number of positive lymph nodes per patient remained consistently low, ranging from 0.18 (Level I) to 0.09 (Level V). These findings suggest that although higher lymph node levels generally yield a greater count of extracted nodes, the proportion testing positive for malignancy diminishes with ascending levels. This trend is illustrated in **Figure 1**.

**Figure 2:**
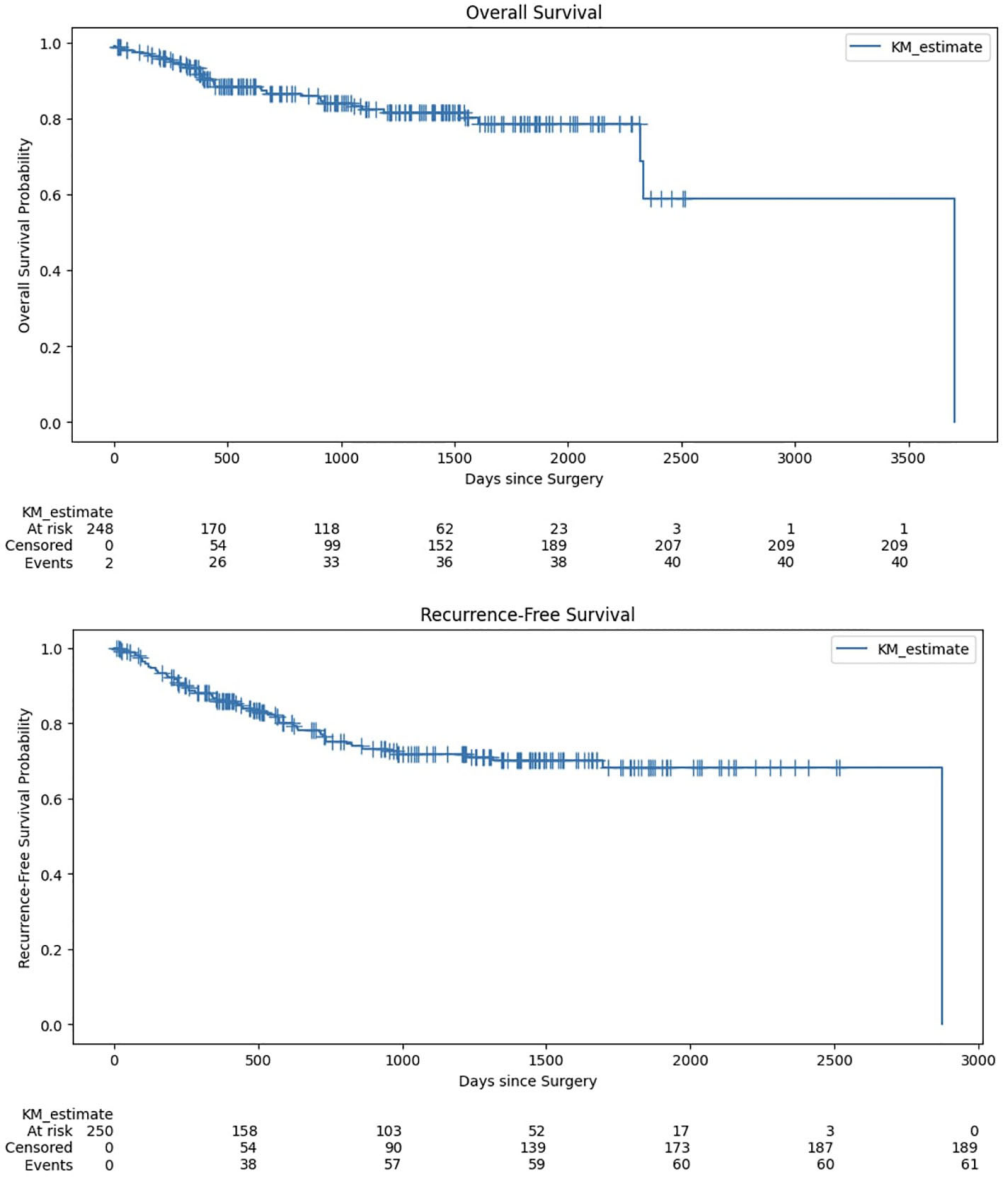
Trends in total number of extracted lymph nodes for each anatomical level (I to V) between 2017-2022 normalized by number of patients.

From 2017 through 2022, the analysis of lymph node resections across five anatomical levels revealed varied trends (**Figure 2**). The total number of lymph nodes resected exhibited fluctuations. The total lymph nodes resected peaked in 2019 with 1,726 nodes, reflecting the most extensive surgical activity within the period. This peak was followed by a sharp decline in 2021, during which only 564 nodes were resected, marking the lowest number of operations in the six-year period. In 2017, the number of lymph nodes resected per level was highest for Level II (11 nodes per patient) and lowest for Level III (3 nodes per patient). In 2018, the total lymph nodes resected per patient remained stable at 31. The highest number of resected nodes was again in Level II (11 nodes per patient). In 2019, there was a decrease in the total lymph nodes resected per patient to 26. The resected nodes per level were relatively consistent with previous years, with Level II remaining the highest (10 nodes per patient). In 2020, the total number of resected lymph nodes per patient increased to 27. The number of resected nodes was highest in Level II (10 nodes per patient). In 2021, there was a moderate increase in the total lymph nodes resected per patient to 28. Level II again had the highest number of resected nodes (12 nodes per patient). In 2022, the total lymph nodes resected per patient increased to 30. The resected nodes per level showed Level II as the highest (12 nodes per patient). Overall, the total number of lymph nodes resected per patient remained relatively stable. The data indicate that Level II consistently had the highest number of resected lymph nodes.

In our analysis, we classified the neck dissection patterns into distinct groups based on the levels of lymph nodes resected. The grouping was predicated on the common surgical practice of defining the scope of neck dissections by the anatomical levels of lymph nodes involved. Specifically, we focused on two primary groups: The “Supraomohyoidal Selective Neck Dissection” (SND) (n=114; 45.60%) group included cases where lymph nodes at Levels I to III were resected, without any extractions at Levels IV and V. This classification aligns with the typical definition of a Supraomohyoidal neck dissection, which targets lymph nodes located above the omohyoid muscle, primarily affected in OSCC. The “Other Dissections” (OD) group (n=136; 54.40%) included all other dissection patterns, which included extractions at Levels IV or V. This group represents more extensive dissection practices.

### 3.3 Number of lymph nodes extracted and length of hospital stay

A significant correlation was observed between the operation time and the number of lymph nodes extracted (Spearman’s rho: 0.519; p < 0.001). A similar significant correlation was found between the number of lymph nodes extracted and the length of hospital stay (Spearman’s rho: 0.284; p < 0.001). Subsequent linear regression analysis, adjusted for multiple factors, revealed that each additional lymph node extracted was significantly associated with an increased hospital stay, specifically an increase of 0.289 days per node (regression coefficient: 0.289; 95% CI: 0.107–0.472; p=0.002). Notably, older age also correlated with prolonged hospitalizations (0.491 days per additional year; 95% CI: 0.228–0.754; p<0.001). Patients with higher T-status (T3 to T4) experienced substantially longer hospital stays compared to those with T0 to T2, with an increase of 13.749 days (95% CI: 6.833–20.666; p<0.001). Furthermore, undergoing a local flap transplant, rather than a microvascular one, was associated with a significantly shorter hospital stay (–20.643 days; 95% CI: –28.196 to –13.090; p<0.001). Interestingly, male sex was also predictive of longer hospital stays (6.372 days; 95% CI: 0.166–12.579; p=0.044). Other factors—including BMI, ASA score, adjuvant therapy, and nodal status—did not demonstrate significant associations with the length of hospital stay (**Table 2**).

**Table 2:** Linear regression model to evaluate the impact of study variables on length of hospital stay.

Model		Sig.	95.0% Confidence Interval for B	
	B		Lower Bound	Upper Bound
BMI	-.429	.169	-1.042	.183
Sex (reference: female)	6.372	.044	.166	12.579
ASA Score (reference: ASA Score 1)	4.123	.154	-1.551	9.797
Transplant (reference: microvascular)	-20.643	<.001	-28.196	-13.090
Age	.491	<.001	.228	.754
Adjuvant therapy (reference: no)	.561	.875	-6.435	7.557
T-status (reference: T1 to T2)	13.749	<.001	6.833	20.666
Nodal-status (reference: N0)	.209	.953	-6.704	7.121
Total number of extracted lymph nodes	.289	.002	.107	.472

### 3.4 Survival analyses

**Figure 3:**
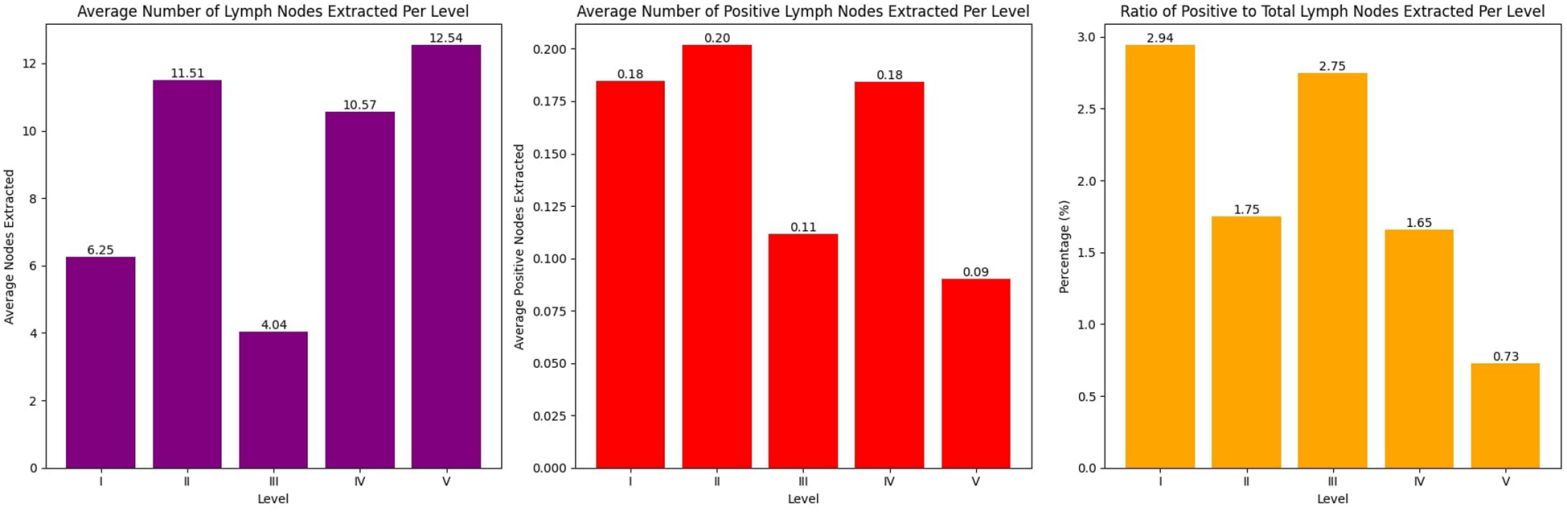
Kaplan-Meier survival analysis for overall survival and recurrence-free survival.

The mean estimate for overall survival, according to the Kaplan-Meier analysis, was 2,781 days with a 95% confidence interval ranging from 2,410.060 to 3,152.754 days. For recurrence-free survival, the mean Kaplan-Meier estimate was 2,129.692 days, with a 95% confidence interval of 1,967.336 to 2,292.049 days (**Figure 3**). The Kaplan-Meier survival analysis for all patients, regardless of dissection group, revealed a 5-year survival probability of 78.68%. The 95% confidence interval for this survival probability ranged from 70.93% to 84.58%.

**Figure 4:**
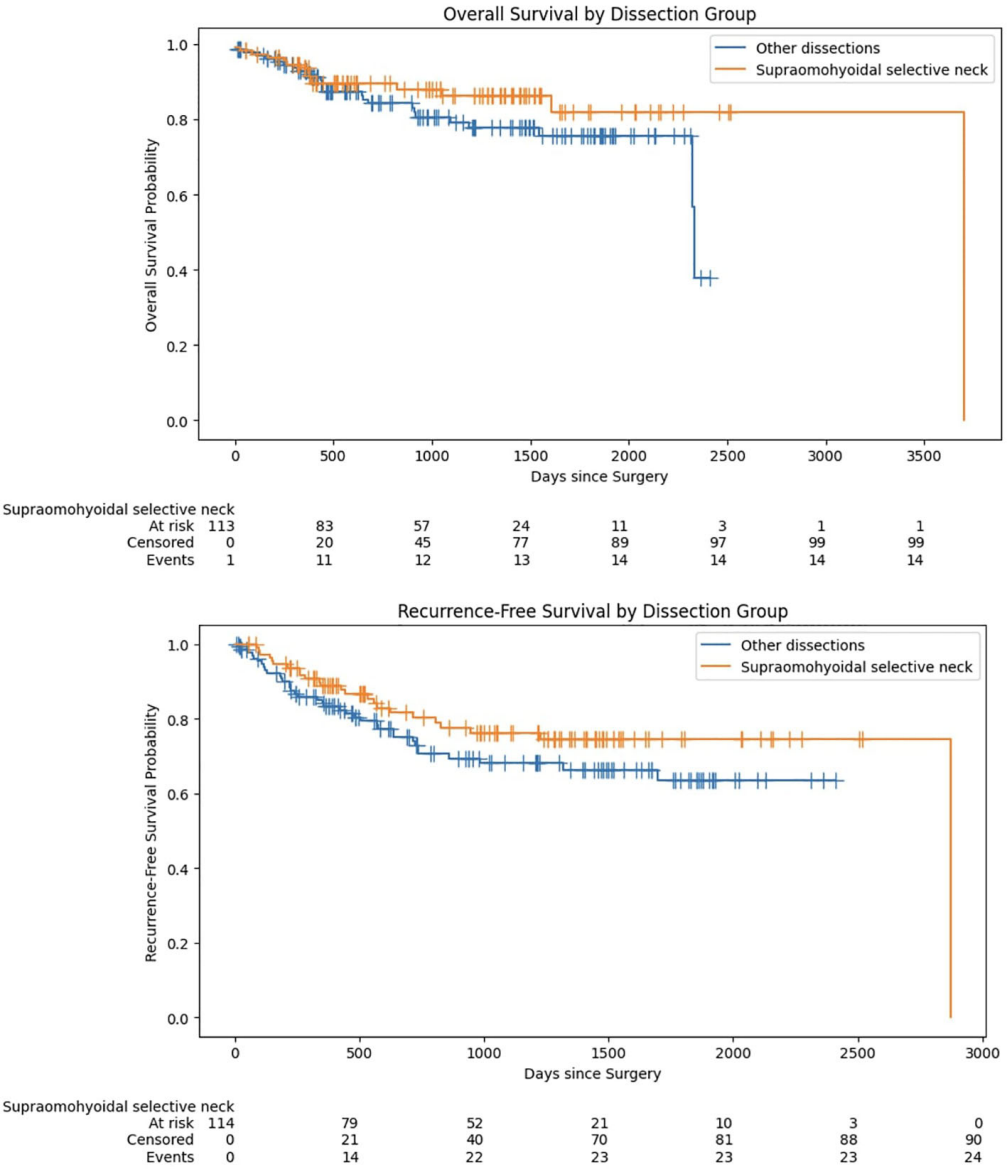
Kaplan-Meier survival analysis for overall survival and recurrence-free survival stratified by lymph node dissection type.

Furthermore, no significant differences were found in the Kaplan-Meier survival analysis when the lymph node dissection groups were treated as factorial variables. Specifically, the log-rank test revealed no notable disparities in overall survival (p=0.153) and recurrence-free survival (p=0.149) between SND and OD (**Figure 4**).

To ensure that the findings regarding survival outcomes were not confounded by demographic, clinical, and treatment-related factors, the analysis controlled for key variables, including sex, age, ASA score, lymph node extraction, and other relevant variables such as adjuvant therapy (see **Tables 3**, **4**). These adjustments were crucial for isolating the effects of surgical techniques and other predictors, thereby providing a clearer understanding of their impact on overall and recurrence-free survival in OSCC.

The Cox regression model for overall survival (**Table 3**) revealed two significant predictors in our cohort. First, age showed a clear impact, with each additional year increasing the risk of mortality by 6.2% (HR = 1.062; 95% CI: 1.026–1.100; p < 0.001). Second, T-status significantly influenced survival: patients with T3/T4 tumors faced roughly 2.8 times the risk of death compared to those with T1/T2 (HR = 2.781; 95% CI: 1.306–5.920; p = 0.008). In contrast, factors such as sex (HR = 2.001; p = 0.081), ASA Score 4 (HR = 6.893; p = 0.195), and the total number of lymph nodes extracted (HR = 0.999; p = 0.959) did not reach statistical significance. Likewise, the neck dissection group (selective vs. other) showed no significant effect on overall survival (p = 0.197). These findings underscore the critical roles of advancing age and higher T-status in driving mortality risk, whereas factors like overall lymph node count were not predictive. Consequently, the previously reported threshold of 18 lymph nodes for selective neck dissections (8) could not be confirmed by our data.

**Table 3:** Results of the Cox regression model for overall survival.

	Sig.	HR	95.0% CI for HR	
			Lower	Upper
BMI	.427	.968	.892	1.049
Sex (reference: female)	.081	2.001	.917	4.363
Operation Time	.496	1.001	.998	1.004
ASA Score 1	Reference			
ASA Score 2	.906	1.134	.139	9.280
ASA Score 3	.644	1.667	.191	14.564
ASA Score 4	.195	6.893	.372	127.657
Transplant (reference: microvascular)	.175	1.923	.748	4.945
Age	<.001	1.062	1.026	1.100
Adjuvant Therapy (reference: no)	.538	1.326	.540	3.259
T-status (reference: T1 to T2)	.008	2.781	1.306	5.920
Nodal-status (reference: N0)	.430	1.334	.652	2.729
Total number of extracted lymph nodes	.959	.999	.975	1.024
Neck Dissection Group (reference: SND)	.197	1.741	.750	4.038

Regarding recurrence-free survival (**Table 4**), age again demonstrated a significant association, with each additional year raising the recurrence risk by 4.6% (HR = 1.046; 95% CI: 1.018–1.076; p = 0.001). Similarly, patients presenting with T3/T4 disease had more than double the risk of recurrence compared to those with T1/T2 (HR = 2.174; 95% CI: 1.187–3.982; p = 0.012). Other variables, including sex, ASA scores, neck dissection group, and the total number of lymph nodes extracted, did not achieve statistical significance. These results highlight the prominence of tumor stage and patient age as key drivers of both overall and recurrence-free survival in our cohort.

**Table 4:** Results of the Cox regression model for recurrence-free survival.

	Sig.	HR	95.0% CI for HR	
			Lower	Upper
BMI	.486	1.020	.965	1.079
Sex (reference: female)	.503	.817	.452	1.477
Operation Time	.465	1.001	.999	1.003
ASA Score 1	Reference			
ASA Score 2	.902	.912	.210	3.960
ASA Score 3	.640	.688	.144	3.301
ASA Score 4	.193	4.102	.490	34.359
Transplant (reference: microvascular)	.343	1.443	.676	3.079
Age	.001	1.046	1.018	1.076
Adjuvant Therapy (reference: no)	.334	1.391	.712	2.718
T-status (reference: T1 to T2)	.012	2.174	1.187	3.982
Nodal-status (reference: N0)	.505	1.234	.664	2.294
Total number of extracted lymph nodes	.323	.990	.971	1.010
Neck Dissection Group (reference: SND)	.064	.921	.844	1.005

To deepen our understanding of these outcomes and identify key predictors for overall and recurrence-free survival, we employed a Cox regression machine learning model designed for survival prediction. The model dedicated to overall survival generated a mean Concordance Index of 0.682 across all folds. Similarly, the model for recurrence-free survival yielded a mean Concordance Index of 0.649 across all five folds. The Concordance Index serves as a measure of the model’s ability to accurately predict the sequence of events—specifically, death and recurrence in this context. Values closer to 1 signify higher predictive accuracy. These results indicate a moderate level of predictive accuracy for survival within our patient cohort using the study variables. The “Number of Positive Lymph Nodes Extracted” emerged as the most significant predictor, underlining its crucial role in estimating mortality risk.

## 4 Discussion

The present study was conducted with the aim of evaluating various aspects of lymph node dissection in OSCC treatment, particularly focusing on the impact of different dissection methods, anatomical levels, and number of lymph nodes extracted on surgical outcomes. The findings are compelling and suggest new opportunities for both clinical practice and future research.

A critical finding of this research is that the extend of lymph node dissection, did not significantly influence the overall survival or recurrence-free survival. The Kaplan-Meier survival analysis, when considering lymph node dissection groups as factorial variables, did not uncover significant differences in overall survival (p=0.153) and recurrence-free survival (p=0.149) between selective neck dissection (SND) and more extensive options (OD). This result is particularly important as it suggests that less invasive or less extensive lymph node dissection methods could offer similar diagnostic and prognostic value as more comprehensive techniques, as suggested before (15, 28, 29). Notably, this finding was not affected by adjuvant therapy. This suggests that the choice of neck dissection method may not necessarily impact survival outcomes directly. It’s important to consider that despite the presence of tumor-positive lymph nodes, which typically predict a poorer prognosis, the choice of a less invasive dissection technique like SND might adequately counterbalance these adverse prognostic implications. This could influence decisions regarding adjuvant therapies, potentially allowing for a tailored approach that reduces the need for more aggressive additional treatments. This nuanced understanding underscores the need for a strategic multidisciplinary selection of surgical and adjuvant therapies based on individual patient profiles rather than a one-size-fits-all approach. This perspective is supported by recent literature (30).

Liu et al. were able to show in a study that the introduction of a multidisciplinary tumor board (MTB) led to a significant survival benefit in patients with head and neck tumors. The benefits of the MTB are multifactorial and improve several elements such as treatment planning and staging (30). The use of adjuvant therapies such as radiotherapy or radiochemotherapy probably plays a crucial role in compensating for the extent of surgery (31). In cases where microscopic tumor remnants remain after SND, these therapies can effectively combat these remnants and thus compensate for a possible survival disadvantage due to the less extensive surgery. Another important parameter is the individual characteristics of the patient, such as health status, concomitant diseases and tumor biology (32, 33). Patients who are more suitable for SND may inherently have a lower tumor stage or fewer risk factors for recurrence. For example, patients with early-stage tumors and negative nodal status may benefit from SND as well as more extensive dissection, as their disease burden is already lower. Conversely, in patients with advanced disease, the efficacy of adjuvant therapies could override the extent of surgery in determining survival prospects. Finally, the type of surgical neck dissection itself could also contribute to these results. SND focuses on removing the lymph nodes most likely to harbor metastases while preserving unaffected structures, which may reduce morbidity without compromising oncological outcomes. When performed accurately and based on sound staging techniques, SND can achieve oncological efficacy comparable to major dissections, especially in well-selected cases (30).

Several contemporary studies have spotlighted the prognostic significance of the “Percentage of Positive Lymph Nodes” over the N status defined by the 8th edition of the AJCC guidelines for TNM status in oral cancer patients (34, 35). The rationale is that the “Percentage of Positive Lymph Nodes” encapsulates not just the N status but the disease’s expanse and the extent of surgical removal of lymph nodes (as quality criterion of surgical expertise). However, when primary lesions transgress the midline, bilateral neck dissection is usually performed. Furthermore, the pathological demarcation between a singular sizable positive node and a confluence of multiple nodes, especially for N3 status, blurs, leading to certain studies eschewing the N3 node status (17, 36, 37). Such hurdles make the “Percentage of Positive Lymph Nodes” utility as a prognostic marker nuanced, contingent on alterations in its components—the positive nodes and the lymph node yield. Kim et al.’s findings in 2011 amplify this perspective, emphasizing that limited neck dissections don’t impede percentage of positive lymph node’s prognostic efficacy (36). It highlights the pertinence of surgical precision over surgical expansiveness (38).

Our study demonstrates a significant variance in the prevalence of tumor-positive lymph nodes across different anatomical levels. Levels I to III had the highest percentages of tumor-positive lymph nodes, despite the most lymph nodes being removed from Levels II and V, as previously reported by other authors (38, 39). Specifically, the high proportion of tumor-positive nodes in Level I underscores the importance of prioritizing this area in surgical procedures. According to Suárez et al., Level IIb is less significant for oral cavity tumors than for oropharyngeal carcinomas, with similar findings for Level IA, depending on the primary tumor location (38, 40). Additionally, the consideration of sparing the XI nerve in Level IIB due to potential nerve damage remains controversial (41). Levels I to III, which yielded the most positive lymph nodes in our study, are encompassed by SND. This finding could allow surgeons to focus their efforts more efficiently, targeting these specific areas for dissection. Notably, studies have described similar results. The majority of authors and also the S3 guideline in Germany describe 20-40% occult metastases precisely in these levels (42–55). Even in the case of metastasis without extranodal growth, the guideline recommends selective neck dissection only up to level III instead of radical dissection up to level V, provided that the dissection is at least one level more caudal than the metastatic level of the LK (56). Another explanation for the significant results in our study for stages I - III could be the lymphatic drainage pathways of the investigated entity. As already mentioned, metastases in OSCC occur most frequently in stages I-III. Candela et al. showed that no level I metastases were found in a patient population of 126 patients with clinical N0 neck dissection. N+ cases showed metastases at this stage in rare cases. However, the authors concluded that surgical dissection of level II-IV seems to be sufficient in these cases, analogous to our results for OSCC and level I-III (57).

Ebrahimi et al.’s 2011 retrospective study examined this relationship, finding that a total lymph node yield exceeding 18 was associated with improved prognosis in patients undergoing SND (58). Subsequent trials echoed this, evidencing enhanced overall survival when using 18 as a threshold in total lymph node yield (7). Typically, more radical procedures retrieve a total lymph node yield surpassing 30 (59). Our cox regression analyses revealed no significant association between overall and recurrence-free survival and the number of lymph nodes extracted. Yet, these findings aren’t uniformly applicable across all oral cancer subsites. For malignancies like tongue or mouth floor cancers, which bear the risk of skip lymph node metastases, more radical interventions remain the gold standard, especially when lymph nodes are confirmed positive (60). Advancements like the sentinel lymph node biopsy for N0 patients emerge as alternatives, curbing the need for invasive procedures and mitigating patient morbidity and economic implications (61). We also found a strong association between the number of lymph nodes extracted and the length of hospital stay. Specifically, each additional lymph node extracted added an extra 0.151 days to the hospital stay, underlining the case for optimizing the surgical process to focus only on necessary lymph node dissections, particularly in the SND levels. In a systematic review it was shown that of 498 patients with oral squamous cell carcinoma who underwent neck lymph node dissection, only 2.8% had positive lymph nodes in Level IV (41). This led to a recommendation for the SND approach, which our results support. These results reflect the trend towards a less invasive and more focused surgical approach as described by several authors (11, 15, 38, 39, 41, 62–65).

Interestingly, the number of lymph nodes removed per patient remained relatively stable from 2017 to 2021, with the exception of deviations caused by the COVID-19 pandemic in view of the fewer operations performed. Heimes et al. examined the impact of COVID-19 lockdowns on oral cavity cancer diagnoses and found that there was no increase in cancer incidence or severity, but shorter intervention times during lockdown. An increase in cases after the lockdown was attributed to dental practice closures, suggesting possible delays in diagnosis (66). Most of the patients in our cohort had a T-status of T1 to T2. Around 40% of patients with OSCC present with limited or early-stage disease, in which treatment is typically single modality, either surgery or radiotherapy (67). Hashim et al. posits that this reduction might be attributed to increased public awareness (68). Yet, they also point out that these positive changes are predominantly seen in high-income countries, whereas low-income nations might exhibit the contrary trend. In this cohort, we further demonstrated that males experience a 2.549-fold increased risk of mortality compared to females (p=0.006), as previously shown. Finally, other factors were found to contribute to the length of hospital stays, such as higher ASA scores, longer operation time, and older age. These predictive factors offer additional context for healthcare planning and could be useful for preoperative patient counseling and perioperative risk stratification (69, 70).

Despite providing valuable insights, our study has several limitations. The relatively small sample size of 250 patients limits the potential for more sophisticated analyses, underscoring the need for larger studies to validate our findings. Further, the retrospective nature of our study inherently introduces potential biases and limitations. Specifically, this design may result in selection bias and confounding variables that could influence the observed outcomes, limiting the ability to establish direct causality between the examined neck dissection techniques and survival outcomes. The association might be influenced by many different factors. However, our results provide valuable insights into the variables analyzed and serve as a foundation for future work. One further limitation of our study is that we did not assess extranodal extension (ENE) in lymph node specimens. ENE+ status has been identified as a powerful prognostic marker in oral squamous cell carcinoma and other head and neck cancers (71). Consequently, our findings regarding the significance of positive lymph nodes may underestimate the magnitude of risk if these nodes also exhibit ENE. Future studies that incorporate ENE status could provide further clarity on the prognostic interplay between lymph node positivity, ENE, and long-term survival outcomes. Another limitation is the absence of an external validation cohort to further assess the generalizability of our findings. Although we employed 5-fold cross-validation as an internal validation method, logistical constraints and the challenges of establishing collaborations with other institutions precluded the inclusion of an independent dataset. Future studies should address this limitation by incorporating external validation cohorts to confirm the robustness and applicability of the model across diverse populations. Another limitation of our study is the exclusion of patients with prior cancer treatments or adjuvant therapies. While these criteria were necessary to create a homogenous cohort and ensure robust analyses, they may limit the generalizability of our findings to OSCC patients with recurrent disease or prior treatments. Future research should consider including these patient groups to evaluate the applicability of the findings to a broader OSCC population.

Expanding the scope of research to include factors such as lymph node density—the ratio of tumor-positive nodes to the total number removed—could offer a more nuanced understanding of prognosis. This parameter has been highlighted in prior studies as a complementary metric to total lymph node counts. Molecular markers, including genomic or proteomic profiles, also hold promise for enhancing predictive accuracy and enabling personalized treatment strategies based on tumor biology. These questions are currently the subject of ongoing research projects and remain unpublished. However, we are planning follow-up studies to explore these avenues, particularly investigating lymph node density and the role of molecular markers in predicting survival and recurrence. These efforts, combined with multi-center, prospective studies, aim to provide a comprehensive framework for optimizing surgical strategies and improving outcomes in OSCC patients. We restricted our inclusion period to 2017-2022 to ensure comparability within the patient collective due to the update of the TNM classification in 2017. Our study specifically focused on OSCC. Given that HPV status is only a minor factor in OSCC, it is not routinely tested in our department and was therefore not considered in our study (72). Another limitation is that the decision for an extended neck dissection was made individually based on the overall clinical context, rather than being subject to standardization. Another limitation is that the decision for an extended neck dissection was made individually based on the overall clinical context, rather than being subject to standardization like for example a frailty screening. Additionally, it should be noted that relatively few lymph nodes are expected in Level III. It is likely that we extended somewhat caudally into Level IV. This suggests that the importance of lymph nodes in Level III may be an artifact arising from an “unclean” boundary with Level IV, as dictated by internal protocols, which limits the generalizability of our results to other cohorts. Although we accounted for known confounders, the retrospective nature of our study necessitates caution regarding other potential confounders. Relevant data such as medication, medical history, or patient compliance were not considered, limiting the final interpretation of our results. Additionally, the cohort was recruited from a single institution and may not represent the broader population, further constraining the generalizability of our findings.

The finding that increased lymph node removal is associated with longer hospital stays highlights the importance of strategic surgical planning in OSCC)management. Each additional lymph node removed correlated with a 0.151-day increase in hospitalization (p < 0.001). This underscores the potential trade-off between extensive dissection and postoperative recovery. While comprehensive lymphadenectomy aims to ensure thorough oncological control, our findings suggest that this approach may lead to prolonged recovery periods, thereby increasing patient morbidity and healthcare costs. This observation supports a more tailored surgical approach, particularly when considering the anatomical levels most likely to harbor tumor-positive nodes. Our analysis identified Levels I to III as critical zones, with Level III specifically associated with improved overall and recurrence-free survival (HR = 0.905 and HR = 0.863, respectively). These findings advocate for focusing on these key levels during neck dissection to maximize oncological outcomes while minimizing unnecessary morbidity associated with more extensive dissections of Levels IV and V, which demonstrated lower positivity rates and limited survival benefits. By optimizing the extent of lymph node removal, surgeons can strike a balance between achieving adequate oncological resection and reducing the physical and logistical burdens of prolonged hospital stays. This aligns with the growing preference for selective neck dissection in OSCC management, particularly for patients without extensive nodal involvement (73). Furthermore, emphasizing a multidisciplinary approach, including preoperative imaging, intraoperative frozen section analysis, and postoperative monitoring, can help refine surgical strategies, ensuring a patient-specific balance between thoroughness and recovery. In conclusion, our findings reinforce the necessity of a personalized approach to lymph node dissection, prioritizing disease-specific nodal levels to optimize survival while mitigating the impact on recovery time. This nuanced perspective is crucial in improving patient outcomes and resource allocation in OSCC treatment.

## 5 Conclusion

Our study into lymph node dissection methods for OSCC treatment revealed that overall survival and recurrence-free survival were not significantly influenced by the specific lymph node dissection technique used or the total number of lymph nodes extracted. This finding suggests that less invasive and more focused procedures could be beneficial, potentially reducing hospital stays and surgical morbidity. We observed a correlation between the number of lymph nodes removed and the length of postoperative hospital stay, highlighting the importance of optimizing the surgical process to focus only on necessary lymph node dissections. Our data underscore the significance of lymph node levels I to III as primary hotspots for tumor-positive nodes, supporting the need for more targeted surgical approaches.

## Data Availability

The raw data supporting the conclusions of this article will be made available by the authors, without undue reservation.

## References

[1] Johnson DE Burtness B Leemans CR Lui VWY Bauman JE Grandis JR. Head and neck squamous cell carcinoma. Nat Rev Dis Primer. (2020) 6:92. doi: 10.1038/s41572-020-00224-3 PMC7944998 33243986

[2] Argiris A Karamouzis MV Raben D Ferris RL. Head and neck cancer. Lancet Lond Engl. (2008) 371:1695–709. doi: 10.1016/S0140-6736(08)60728-X PMC7720415 18486742

[3] Anderson G Ebadi M Vo K Novak J Govindarajan A Amini A. An updated review on head and neck cancer treatment with radiation therapy. Cancers. (2021) 13:4912. doi: 10.3390/cancers13194912 34638398 PMC8508236

[4] Bonner JA Harari PM Giralt J Azarnia N Shin DM Cohen RB. Radiotherapy plus cetuximab for squamous-cell carcinoma of the head and neck. N Engl J Med. (2006) 354:567–78. doi: 10.1056/NEJMoa053422 16467544

[5] Graboyes EM Gross J Kallogjeri D Piccirillo JF Al-Gilani M Stadler ME. Association of compliance with process-related quality metrics and improved survival in oral cavity squamous cell carcinoma. JAMA Otolaryngol Neck Surg. (2016) 142:430–7. doi: 10.1001/jamaoto.2015.3595 PMC6086583 26869135

[6] Ebrahimi A Clark JR Zhang WJ Elliott MS Gao K Milross CG. Lymph node ratio as an independent prognostic factor in oral squamous cell carcinoma. Head Neck. (2011) 33:1245–51. doi: 10.1002/hed.21600 20967874

[7] Divi V Harris J Harari PM Cooper JS McHugh J Bell D. Establishing quality indicators for neck dissection: Correlating the number of lymph nodes with oncologic outcomes (NRG Oncology RTOG 9501 and RTOG 0234). Cancer. (2016) 122:3464–71. doi: 10.1002/cncr.30204 PMC5237619 27419843

[8] Foo CC Ku C Wei R Yip J Tsang J Chan TY. How does lymph node yield affect survival outcomes of stage I and II colon cancer? World J Surg Oncol. (2020) 18:22. doi: 10.1186/s12957-020-1802-6 31996214 PMC6990535

[9] Rosenberger LH Ren Y Thomas SM Greenup RA Fayanju OM Hwang ES. Axillary lymph node dissection in node-positive breast cancer: are ten nodes adequate and when is enough, enough? Breast Cancer Res Treat. (2020) 179:661–70. doi: 10.1007/s10549-019-05500-9 PMC7049074 31741179

[10] López F Fernández-Vañes L García-Cabo P Grilli G Álvarez-Marcos C Llorente JL. Selective neck dissection in the treatment of head and neck squamous cell carcinoma patients with a clinically positive neck. Oral Oncol. (2020) 102:104565. doi: 10.1016/j.oraloncology.2020.104565 31945661

[11] Sheppard SC Frech L Giger R Nisa L. Lymph node yield and ratio in selective and modified radical neck dissection in head and neck cancer—Impact on oncological outcome. Cancers. (2021) 13:2205. doi: 10.3390/cancers13092205 34064344 PMC8125696

[12] Andersen PE Warren F Spiro J Burningham A Wong R Wax MK. Results of selective neck dissection in management of the node-positive neck. Arch Otolaryngol Head Neck Surg. (2002) 128:1180–4. doi: 10.1001/archotol.128.10.1180 12365890

[13] Cheraghlou S Otremba M Kuo Yu P Agogo GO Hersey D Judson BL. Prognostic value of lymph node yield and density in head and neck Malignancies. Otolaryngol Neck Surg. (2018) 158:1016–23. doi: 10.1177/0194599818756830 29460685

[14] Deutschmann MW Chin-Lenn L Au J Brilz A Nakoneshny S Dort JC. Extent of central neck dissection among thyroid cancer surgeons: Cross-sectional analysis. Head Neck. (2016) 38 Suppl 1:E328–332. doi: 10.1002/hed.23996 25546489

[15] Divi V Chen MM Nussenbaum B Rhoads KF Sirjani DB Holsinger FC. Lymph node count from neck dissection predicts mortality in head and neck cancer. J Clin Oncol Off J Am Soc Clin Oncol. (2016) 34:3892–7. doi: 10.1200/JCO.2016.67.3863 27480149

[16] Voss JO Freund L Neumann F Mrosk F Rubarth K Kreutzer K. Prognostic value of lymph node involvement in oral squamous cell carcinoma. Clin Oral Investig. (2022) 26:6711–20. doi: 10.1007/s00784-022-04630-7 PMC9643253 35895143

[17] Ong W Zhao R Lui B Tan W Ebrahimi A Clark JR. Prognostic significance of lymph node density in squamous cell carcinoma of the tongue. Head Neck. (2016) 38 Suppl 1:E859–866. doi: 10.1002/hed.24113 25917601

[18] Jin W Zhu Z Wu Y Ding X Wu H Song X. Prognostic value of log odds of positive lymph nodes in patients with resectable oral squamous cell carcinoma. Oral Oncol. (2020) 108:104709. doi: 10.1016/j.oraloncology.2020.104709 32535340

[19] Künzel J Mantsopoulos K Psychogios G Agaimy A Grundtner P Koch M. Lymph node ratio is of limited value for the decision-making process in the treatment of patients with laryngeal cancer. Eur Arch Oto-Rhino-Laryngol Off J Eur Fed Oto-Rhino-Laryngol Soc EUFOS Affil Ger Soc Oto-Rhino-Laryngol - Head Neck Surg. (2015) 272:453–61. doi: 10.1007/s00405-014-2997-3 24643852

[20] Suresh GM Koppad R Prakash BV Sabitha KS Dhara PS. Prognostic indicators of oral squamous cell carcinoma. Ann Maxillofac Surg. (2019) 9:364–70. doi: 10.4103/ams.ams_253_18 PMC6933976 31909017

[21] Sharma K Ahlawat P Gairola M Tandon S Sachdeva N Sharief MI. Prognostic factors, failure patterns and survival analysis in patients with resectable oral squamous cell carcinoma of the tongue. Radiat Oncol J. (2019) 37:73–81. doi: 10.3857/roj.2018.00577 31266288 PMC6610009

[22] Huang TH Li KY Choi WS. Lymph node ratio as prognostic variable in oral squamous cell carcinomas: Systematic review and meta-analysis. Oral Oncol. (2019) 89:133–43. doi: 10.1016/j.oraloncology.2018.12.032 30732951

[23] Ferreira AKA de Carvalho SHG Granville-Garcia AF Sarmento DJ de S Agripino GG de Abreu MHNG. Survival and prognostic factors in patients with oral squamous cell carcinoma. Med Oral Patol Oral Cir Bucal. (2021) 26:e387–92. doi: 10.4317/medoral.24242 PMC8141315 33037796

[24] Oliveira LL Bergmann A Melo AC Thuler LCS. Prognostic factors associated with overall survival in patients with oral cavity squamous cell carcinoma. Med Oral Patol Oral Cir Bucal. (2020) 25:e523–31. doi: 10.4317/medoral.23558 PMC7338068 32520923

[25] von Elm E Altman DG Egger M Pocock SJ Gøtzsche PC Vandenbroucke JP. The Strengthening the Reporting of Observational Studies in Epidemiology (STROBE) statement: guidelines for reporting observational studies. J Clin Epidemiol. (2008) 61:344–9. doi: 10.1016/j.jclinepi.2007.11.008 18313558

[26] Lydiatt WM Patel SG O’Sullivan B Brandwein MS Ridge JA Migliacci JC. Head and neck cancers—major changes in the American Joint Committee on cancer eighth edition cancer staging manual. CA Cancer J Clin. (2017) 67:122–37. doi: 10.3322/caac.21389 28128848

[27] Struckmeier A-K Eichhorn P Agaimy A Buchbender M Moest T Lutz R. Comparison of the 7th and revised 8th UICC editions (2020) for oral squamous cell carcinoma: How does the reclassification impact staging and survival? Virchows Arch. (2024) 484:901–13. doi: 10.1007/s00428-023-03727-y PMC11186894 38191928

[28] Rodrigo JP Grilli G Shah JP Medina JE Robbins KT Takes RP. Selective neck dissection in surgically treated head and neck squamous cell carcinoma patients with a clinically positive neck: Systematic review. Eur J Surg Oncol. (2018) 44:395–403. doi: 10.1016/j.ejso.2018.01.003 29395434

[29] Fasunla AJ Greene BH Timmesfeld N Wiegand S Werner JA Sesterhenn AM. A meta-analysis of the randomized controlled trials on elective neck dissection versus therapeutic neck dissection in oral cavity cancers with clinically node-negative neck. Oral Oncol. (2011) 47:320–4. doi: 10.1016/j.oraloncology.2011.03.009 21459661

[30] Liu JC Kaplon A Blackman E Miyamoto C Savior D Ragin C. The impact of the multidisciplinary tumor board on head and neck cancer outcomes. Laryngoscope. (2020) 130:946–50. doi: 10.1002/lary.28066 PMC7868105 31095740

[31] Liao C-T Kang C-J Lee L-Y Hsueh C Lin C-Y Fan K-H. Association between multidisciplinary team care approach and survival rates in patients with oral cavity squamous cell carcinoma. Head Neck. (2016) 38 Suppl 1:E1544–1553. doi: 10.1002/hed.24276 26890807

[32] Prades J Remue E van Hoof E Borras JM. Is it worth reorganising cancer services on the basis of multidisciplinary teams (MDTs)? A systematic review of the objectives and organisation of MDTs and their impact on patient outcomes. Health Policy Amst Neth. (2015) 119:464–74. doi: 10.1016/j.healthpol.2014.09.006 25271171

[33] Wheless SA McKinney KA Zanation AM. A prospective study of the clinical impact of a multidisciplinary head and neck tumor board. Otolaryngol–Head Neck Surg Off J Am Acad Otolaryngol-Head Neck Surg. (2010) 143:650–4. doi: 10.1016/j.otohns.2010.07.020 PMC2994101 20974334

[34] Gartagani Z Doumas S Kyriakopoulou A Economopoulou P Psaltopoulou T Kotsantis I. Lymph node ratio as a prognostic factor in neck dissection in oral cancer patients: A systematic review and meta-analysis. Cancers. (2022) 14:4456. doi: 10.3390/cancers14184456 36139617 PMC9497248

[35] Reinisch S Kruse A Bredell M Lübbers H-T Gander T Lanzer M. Is lymph-node ratio a superior predictor than lymph node status for recurrence-free and overall survival in patients with head and neck squamous cell carcinoma? Ann Surg Oncol. (2014) 21:1912–8. doi: 10.1245/s10434-014-3634-5 24652351

[36] Kim SY Nam SY Choi S-H Cho K-J Roh J-L. Prognostic value of lymph node density in node-positive patients with oral squamous cell carcinoma. Ann Surg Oncol. (2011) 18:2310–7. doi: 10.1245/s10434-011-1614-6 21336511

[37] Bharath VM Balagopal PG Nebu AG Jayasudha AV Iqbal Ahmed M Sebastian P. Can Metastatic Lymph node ratio be used as an independent prognostic factor in Carcinoma tongue? Gulf J Oncolog. (2018) 1:6–10. 30344127

[38] Talmi YP Takes RP Alon EE Nixon IJ López F de Bree R. Prognostic value of lymph node ratio in head and neck squamous cell carcinoma. Head Neck. (2018) 40:1082–90. doi: 10.1002/hed.25080 29394461

[39] Ferlito A Silver CE Rinaldo A. Elective management of the neck in oral cavity squamous carcinoma: current concepts supported by prospective studies. Br J Oral Maxillofac Surg. (2009) 47:5–9. doi: 10.1016/j.bjoms.2008.06.001 19121878

[40] Suárez C Rodrigo JP Robbins KT Paleri V Silver CE Rinaldo A. Superselective neck dissection: rationale, indications, and results. Eur Arch Otorhinolaryngol. (2013) 270:2815–21. doi: 10.1007/s00405-012-2344-5 23321797

[41] Shabtay NW Ronen O. Level IV neck dissection as an elective treatment for oral tongue carcinoma—a systematic review and meta-analysis. Oral Surg Oral Med Oral Pathol Oral Radiol. (2020) 130:363–72. doi: 10.1016/j.oooo.2020.04.810 32540318

[42] Wenig BM. Squamous cell carcinoma of the upper aerodigestive tract: dysplasia and select variants. Mod Pathol. (2017) 30:S112–8. doi: 10.1038/modpathol.2016.207 28060368

[43] Byers RM El-Naggar AK Lee YY Rao B Fornage B Terry NH. Can we detect or predict the presence of occult nodal metastases in patients with squamous carcinoma of the oral tongue? Head Neck. (1998) 20:138–44. doi: 10.1002/(sici)1097-0347(199803)20:2<138::aid-hed7>3.0.co;2-3 9484945

[44] Spiro RH Morgan GJ Strong EW Shah JP. Supraomohyoid neck dissection. Am J Surg. (1996) 172:650–3. doi: 10.1016/s0002-9610(96)00300-5 8988669

[45] Nieuwenhuis EJC Castelijns JA Pijpers R van den Brekel MWM Brakenhoff RH van der Waal I. Wait-and-see policy for the N0 neck in early-stage oral and oropharyngeal squamous cell carcinoma using ultrasonography-guided cytology: is there a role for identification of the sentinel node? Head Neck. (2002) 24:282–9. doi: 10.1002/hed.10018 11891961

[46] Shah JP Candela FC Poddar AK. The patterns of cervical lymph node metastases from squamous carcinoma of the oral cavity. Cancer. (1990) 66:109–13. doi: 10.1002/1097-0142(19900701)66:1<109::aid-cncr2820660120>3.0.co;2-a 2354399

[47] Spiro JD Spiro RH Shah JP Sessions RB Strong EW. Critical assessment of supraomohyoid neck dissection. Am J Surg. (1988) 156:286–9. doi: 10.1016/s0002-9610(88)80293-9 3177752

[48] Byers RM Wolf PF Ballantyne AJ. Rationale for elective modified neck dissection. Head Neck Surg. (1988) 10:160–7. doi: 10.1002/hed.2890100304 3235344

[49] Crean S-J Hoffman A Potts J Fardy MJ. Reduction of occult metastatic disease by extension of the supraomohyoid neck dissection to include level IV. Head Neck. (2003) 25:758–62. doi: 10.1002/hed.10282 12953312

[50] Hao S-P Tsang NM. The role of supraomohyoid neck dissection in patients of oral cavity carcinoma. Oral Oncol. (2002) 38:309–12. doi: 10.1016/s1368-8375(01)00061-6 11978555

[51] O’Brien CJ Traynor SJ McNeil E McMahon JD Chaplin JM. The use of clinical criteria alone in the management of the clinically negative neck among patients with squamous cell carcinoma of the oral cavity and oropharynx. Arch Otolaryngol Neck Surg. (2000) 126:360–5. doi: 10.1001/archotol.126.3.360 10722009

[52] Turner SL Slevin NJ Gupta NK Swindell R. Radical external beam radiotherapy for 333 squamous carcinomas of the oral cavity–evaluation of late morbidity and a watch policy for the clinically negative neck. Radiother Oncol J Eur Soc Ther Radiol Oncol. (1996) 41:21–9. doi: 10.1016/s0167-8140(96)91785-5 8961364

[53] Henick DH Silver CE Heller KS Shaha AR El GH Wolk DP. Supraomohyoid neck dissection as a staging procedure for squamous cell carcinomas of the oral cavity and oropharynx. Head Neck. (1995) 17:119–23. doi: 10.1002/hed.2880170208 7558808

[54] McGuirt WF Johnson JT Myers EN Rothfield R Wagner R. Floor of mouth carcinoma. The management of the clinically negative neck. Arch Otolaryngol Head Neck Surg. (1995) 121:278–82. doi: 10.1001/archotol.1995.01890030020004 7873143

[55] Kligerman J Lima RA Soares JR Prado L Dias FL Freitas EQ. Supraomohyoid neck dissection in the treatment of T1/T2 squamous cell carcinoma of oral cavity. Am J Surg. (1994) 168:391–4. doi: 10.1016/s0002-9610(05)80082-0 7977957

[56] Leitlinienprogramm Onkologie (Deutsche Krebsgesellschaft, Deutsche Krebshilfe, AWMF): S3-Leitlinie Diagnostik und Therapie des Mundhöhlenkarzinoms, Langversion 3.0, 2021, AWMF Registernummer: 007/100OL. Available at: https://register.awmf.org/assets/guidelines/007-100OLl_S3-Diagnostik-Therapie-Mundhoehlenkarzinom_2021-03.pdf (Accessed April 11, 2025).

[57] Candela FC Kothari K Shah JP. Patterns of cervical node metastases from squamous carcinoma of the oropharynx and hypopharynx. Head Neck. (1990) 12:197–203. doi: 10.1002/hed.2880120302 2358329

[58] Ebrahimi A Zhang WJ Gao K Clark JR. Nodal yield and survival in oral squamous cancer: Defining the standard of care. Cancer. (2011) 117:2917–25. doi: 10.1002/cncr.25834 21246523

[59] Friedman M Lim JW Dickey W Tanyeri H Kirshenbaum GL Phadke DM. Quantification of lymph nodes in selective neck dissection. Laryngoscope. (1999) 109:368–70. doi: 10.1097/00005537-199903000-00005 10089959

[60] Omura K. Current status of oral cancer treatment strategies: surgical treatments for oral squamous cell carcinoma. Int J Clin Oncol. (2014) 19:423–30. doi: 10.1007/s10147-014-0689-z 24682763

[61] Monroe MM Lai SY. Sentinel lymph node biopsy for oral cancer: supporting evidence and recent novel developments. Curr Oncol Rep. (2014) 16:385. doi: 10.1007/s11912-014-0385-1 24633882

[62] Murer K Huber GF Haile SR Stoeckli SJ. Comparison of morbidity between sentinel node biopsy and elective neck dissection for treatment of the n0 neck in patients with oral squamous cell carcinoma. Head Neck. (2011) 33:1260–4. doi: 10.1002/hed.21622 21837694

[63] D’Cruz AK Vaish R Kapre N Dandekar M Gupta S Hawaldar R. Elective versus therapeutic neck dissection in node-negative oral cancer. N Engl J Med. (2015) 373:521–9. doi: 10.1056/NEJMoa1506007 26027881

[64] Ding D Stokes W Eguchi M Hararah M Sumner W Amini A. Association between lymph node ratio and recurrence and survival outcomes in patients with oral cavity cancer. JAMA Otolaryngol– Head Neck Surg. (2019) 145:53–61. doi: 10.1001/jamaoto.2018.2974 30452499 PMC6439806

[65] Prabhu RS Hanasoge S Magliocca KR Hall WA Chen SA Higgins KA. Lymph node ratio influence on risk of head and neck cancer locoregional recurrence after initial surgical resection: implications for adjuvant therapy. Head Neck. (2015) 37:777–82. doi: 10.1002/hed.23662 24596123

[66] Heimes D Müller LK Schellin A Naujokat H Graetz C Schwendicke F. Consequences of the COVID-19 pandemic and governmental containment policies on the detection and therapy of oral Malignant lesions-A retrospective, multicenter cohort study from Germany. Cancers. (2021) 13:2892. doi: 10.3390/cancers13122892 34207863 PMC8227890

[67] Lee Y-G Kang EJ Keam B Choi J-H Kim J-S Park KU. Treatment strategy and outcomes in locally advanced head and neck squamous cell carcinoma: a nationwide retrospective cohort study (KCSG HN13–01). BMC Cancer. (2020) 20:813. doi: 10.1186/s12885-020-07297-z 32854649 PMC7450571

[68] Hashim D Genden E Posner M Hashibe M Boffetta P. Head and neck cancer prevention: from primary prevention to impact of clinicians on reducing burden. Ann Oncol. (2019) 30:744–56. doi: 10.1093/annonc/mdz084 PMC6551449 30840052

[69] Kudulaiti N Zhou Z Luo C Zhang J Zhu F Wu J. A nomogram for individualized prediction of overall survival in patients with newly diagnosed glioblastoma: a real-world retrospective cohort study. BMC Surg. (2021) 21:238. doi: 10.1186/s12893-021-01233-z 33957923 PMC8101102

[70] Ao L Shi D Liu D Yu H Xu L Xia Y. A survival nomogram model constructed with common clinical characteristics to assist clinical decisions for diffuse low-grade gliomas: A population analysis based on SEER database. Front Oncol. (2023) 13:963688. doi: 10.3389/fonc.2023.963688 36845716 PMC9947492

[71] Carta F Quartu D Mariani C Tatti M Marrosu V Gioia E. Compartmental surgery with microvascular free flap reconstruction in patients with T1-T4 squamous cell carcinoma of the tongue: analysis of risk factors, and prognostic value of the 8th edition AJCC TNM staging system. Front Oncol. (2020) 10:984. doi: 10.3389/fonc.2020.00984 32760667 PMC7372302

[72] Fonsêca TC Jural LA Marañón-Vásquez GA Magno MB Roza ALOC Ferreira DMTP. Global prevalence of human papillomavirus-related oral and oropharyngeal squamous cell carcinomas: a systematic review and meta-analysis. Clin Oral Investig. (2023) 28:62. doi: 10.1007/s00784-023-05425-0 38158517

[73] Werner JA Dünne AA Myers JN. Functional anatomy of the lymphatic drainage system of the upper aerodigestive tract and its role in metastasis of squamous cell carcinoma. Head Neck. (2003) 25:322–32. doi: 10.1002/hed.10257 12658737

